# Systematic review on the effectiveness of augmented reality applications in medical training

**DOI:** 10.1007/s00464-016-4800-6

**Published:** 2016-02-23

**Authors:** E. Z. Barsom, M. Graafland, M. P. Schijven

**Affiliations:** 1 Department of Surgery, Academic Medical Centre, PO Box 22660, 1100 DD Amsterdam, The Netherlands; 2 Department of Surgery, Flevo Hospital, Almere, The Netherlands

**Keywords:** Augmented reality, Training, Medical specialist training, Surgery, Medical education

## Abstract

**Background:**

Computer-based applications are increasingly used to support the training of medical professionals. Augmented reality applications (ARAs) render an interactive virtual layer on top of reality. The use of ARAs is of real interest to medical education because they blend digital elements with the physical learning environment. This will result in new educational opportunities. The aim of this systematic review is to investigate to which extent augmented reality applications are currently used to validly support medical professionals training.

**Methods:**

PubMed, Embase, INSPEC and PsychInfo were searched using predefined inclusion criteria for relevant articles up to August 2015. All study types were considered eligible. Articles concerning AR applications used to train or educate medical professionals were evaluated.

**Results:**

Twenty-seven studies were found relevant, describing a total of seven augmented reality applications. Applications were assigned to three different categories. The first category is directed toward laparoscopic surgical training, the second category toward mixed reality training of neurosurgical procedures and the third category toward training echocardiography. Statistical pooling of data could not be performed due to heterogeneity of study designs. Face-, construct- and concurrent validity was proven for two applications directed at laparoscopic training, face- and construct validity for neurosurgical procedures and face-, content- and construct validity in echocardiography training. In the literature, none of the ARAs completed a full validation process for the purpose of use.

**Conclusion:**

Augmented reality applications that support blended learning in medical training have gained public and scientific interest. In order to be of value, applications must be able to transfer information to the user. Although promising, the literature to date is lacking to support such evidence.

Simulation of critical situations creates a promising opportunity for the education of medical professionals in a safe environment [1]. Virtual reality (VR) modalities may create a digital environment, designed to resemble aspects of the real world. As a result, trainees using VR simulation learn tasks in a setting closely mimicking relevant realistic situations. Relevant scenarios can thus be practiced in surroundings where exploration and troubleshooting are safe. Applications using VR have shown to be able to improve learning outcome for different training procedures for various medical specialists [2–5]. Much desired outcomes in healthcare such as improvement of patient safety and the reduction in costs and morbidity after use of computer-enhanced training have been reported [6].

Caudell introduced the term ‘augmented reality’ (AR) in 1990 while working for Boeings Computer Services [7]. Workers were guided through the use of a head-mounted display to perform electrical wiring for aircraft equipment, without having to interpret abstract diagrams in manuals, allowing performing tasks without hours of effort to study [8]. In medicine, complex sequential tasks must be mastered; number of operations and quality maintained, while working hours are reduced [9–11]. Whilst conditions at the workplace for learning in terms of hours and opportunities are under stress, adequate training experiences must be ensured.

VR refers to a digital environment in which the user interacts as if it takes place in the real world. However, the focus of the interaction remains in the digital environment. AR differs from VR because the focus of the interaction of the performed task lies within in the real world (AR) instead of the digital environment (VR). AR thus offers the opportunity of a digital, often interactive overlay onto a real or virtual environment. Augmented reality applications (ARAs) are digital applications offering such an extra layer. To the user, layers of the virtual and physical environment are blended in such a way that an immersive, interactive environment is experienced. Hence, ARAs may have great potential in training medical personnel.

Modern teaching curricula aim to educate trainees efficiently and in a safe environment. Educational methods currently being used in medical specialist training include practice-based learning, problem-based learning [12, 13] team-based learning [14, 15], eLearning [16, 17] and (VR) simulation training [1]. Although VR learning environments offer opportunities for full- and partial-task training, they are often a mere representation of a task in reality [18]. This may result in medical specialists that may be well trained for a particular task on the job in a set context, but who lack competencies needed to adapt to ever-changing situations in the real working environment [19]. To acquire stable, crossover competencies, it is necessary to create a training environment offering flexibility and adaptation in training true-to-life working processes in changing environments as is much needed in medical settings. As medical specialist training involves complex learning [20], ARAs are of great potential.

Within healthcare, ARAs have been developed to train or educate medical professionals [21], as a navigation tool during surgical procedures [22, 23] to enhance visualization at the operating room [24] and as a therapeutic tool in the treatment of patients [25–27].

The aim of this review is to identify the value of ARAs for training professionals in medicine. The first objective is to provide an overview of ARAs used in medical training. The second objective is to evaluate their validity in doing so systematically.

## Methods

### Search criteria

A systematic literature search was performed in search of reports using ARAs to train or educate medical professionals validly. For our search, we classified ARAs as systems that use digital content in combination with real-time user interaction, tied to a specific time and location, resulting in a computer-based enhancement of the real environment [28]. A training tool was defined as an application aimed at improvement of performance or skills. A medical professional refers to an individual taking care of patients in an institutionalized setting, or in formal training to do so. Reports addressing VR without AR components were excluded from analysis.

### Study selection and assessment AR applications

PubMed, Embase, INSPEC and PsychInfo were searched for key terms (medical or surgery) AND (augmented reality) AND (educat* OR simulat* OR training). The latest search was conducted on August 28, 2015. All study types were considered eligible for inclusion. Reports that did not relate to a learning context for medical professionals were excluded from analysis, as were conference proceedings, reviews and studies investigating internal validity or technological aspects. All reports were screened on title and abstract according to the aforementioned criteria. Reports deemed ‘relevant,’ ‘dubious’ or ‘unknown’ were examined in full text. The reference lists of the reports assessed for eligibility were searched for other relevant reports. None of the reports were excluded because of language. The Internet was searched, and study authors were contacted directly in case of incompleteness of the data in a report. The following data were extracted from all reports: name, system, purpose, target group and validity evidence.

### Review of studies

All methods developed for the training and education of medical professionals should be assessed for their validity according to several consensus criteria [29, 30]. A validation process encompasses multiple interrelated stages, which all investigate the ability of the training instrument to improve or measure the construct it is intended to improve or measure (Table 1) [30]. To evaluate the degree to which an ARA resembles the real working situation, experts and novices were required to assess ARAs resemblance with the situation (*face validity*). The *content validity* of an ARA relates to a uniform and positive evaluation of the educational content by subjects considered to be experts in the field. *Construct validity* is defined as the degree to which results of a training session as performed by the trainee using the ARA reflect the actual skill of the trainee who is being assessed [28, 30]. *Concurrent validity* refers to performance improvement using the ARA compared to an established training method (gold standard). Finally, to ensure that professionals are not only well trained in an AR environment, but that this skill also translates to the real world, predictive validity of the ARA must be assessed. These steps comprise a full validation process. Only if all parts of this process have been positively evaluated, sufficient proof has been gathered for the training instrument to be implemented in practice.

**Table 1:** Matrix of validity type for augmented reality applications (ARA) to train or educate medical professionals

Stages of validity	Description	Criteria for achievement	Appropriate method of examination
1. Face validity	The degree of resemblance between an ARA and the educational construct as assessed by medical experts (referents) and novices (trainees)	Uniform and positive evaluation of the resemblance between the ARA with the educational construct among novice and expert medical professionals	Questionnaire after use of the ARA
2. Content validity	The degree to which the ARA content adequately covers the dimensions of the medical content it aims to educate (or is associated with) (‘the truth whole truth and nothing but the truth’)	Uniform and positive evaluation of the ARA content and associated testing parameters by panel considered to be experts in the field	Questionnaire considering the content of the ARA
3. Construct validity	Inherent difference in outcome between experts and novices on outcome parameters relevant to the educational construct	Outcome differences considered to be of statistical significance between subjects considered to be of different levels of skill	Comparative study measuring the relevant outcome parameters on the ARA for subjects with presumed different levels of expertise in the educational construct.
4. Concurrent validity	Concordance of subject outcome parameters using tie ARA compared to outcome parameters on an established instrument or method, believed to measure the same educational construct (preferably the golden standard) training method)	Study results show correlation considered to be significant between ARA and the alternative, established training method	Comparative study comparing the outcome parameters of two different training methods in the same study participants
5. Predictive validity	The degree of concordance of ARA outcome parameters and subjects’ performance on the educational construct it aims to resemble in reality	Metrics show correlation considered to be significant between relevant outcome parameters on ARA and performance on educational construct it aims to resemble in reality	Randomized controlled trial comparing performance on educational construct in reality before/after training on ARA and control group using another training method

Data extraction on validity studies was in accordance with the *Cochrane Handbook for Systematic Reviews of Interventions* [31] and concerned methodological aspects (study design, intention to treat, randomization, concealment of allocation, blinding, follow-up and other possible bias), details of the ARA, details on intervention, primary and secondary endpoints, instruments, timing, results of measurements performed and funding. Quality of the randomized controlled trials was systematically assessed using the Cochrane Collaboration’s tool for assessing risk of bias, estimating the level of risk being either high or low. The methodological index for non-randomized studies (MINORS) was used to assess the quality of observational studies. This instrument uses a 12-item scale, scoring a maximum score of 16 points for non-comparative studies and 24 for comparative studies [32]. The articles were rated according to a modified form of the Oxford Centre for Evidence-Based Medicine (CEBM). The data extracted was used to assess the validation steps achieved in a validation process. Two reviewers extracted data independently, and in case of disagreement, a third reviewer was consulted.

## Results

**Fig. 1:**
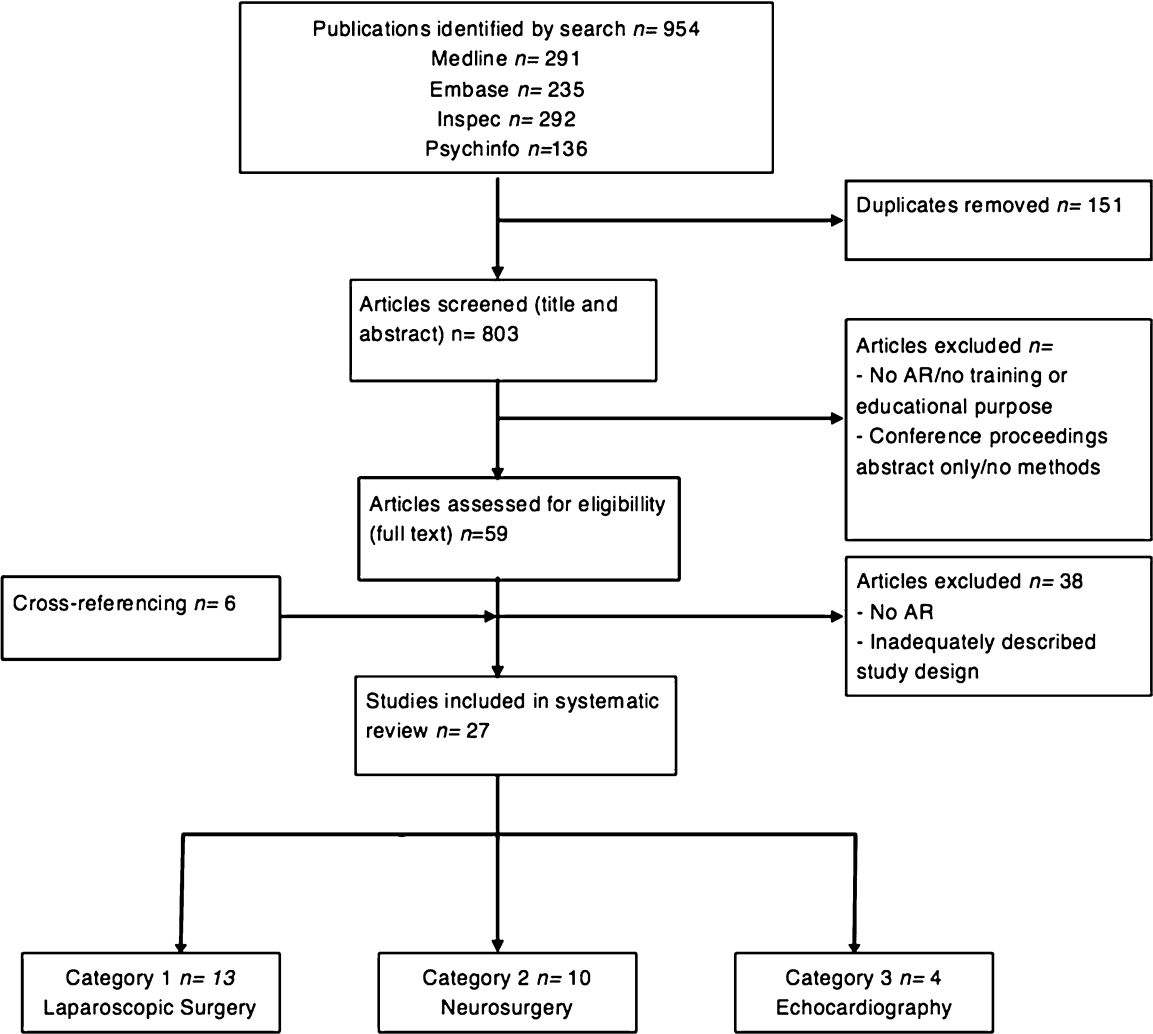
Search strategy on augmented reality applications to train or educate medical professionals

**Fig. 2:**
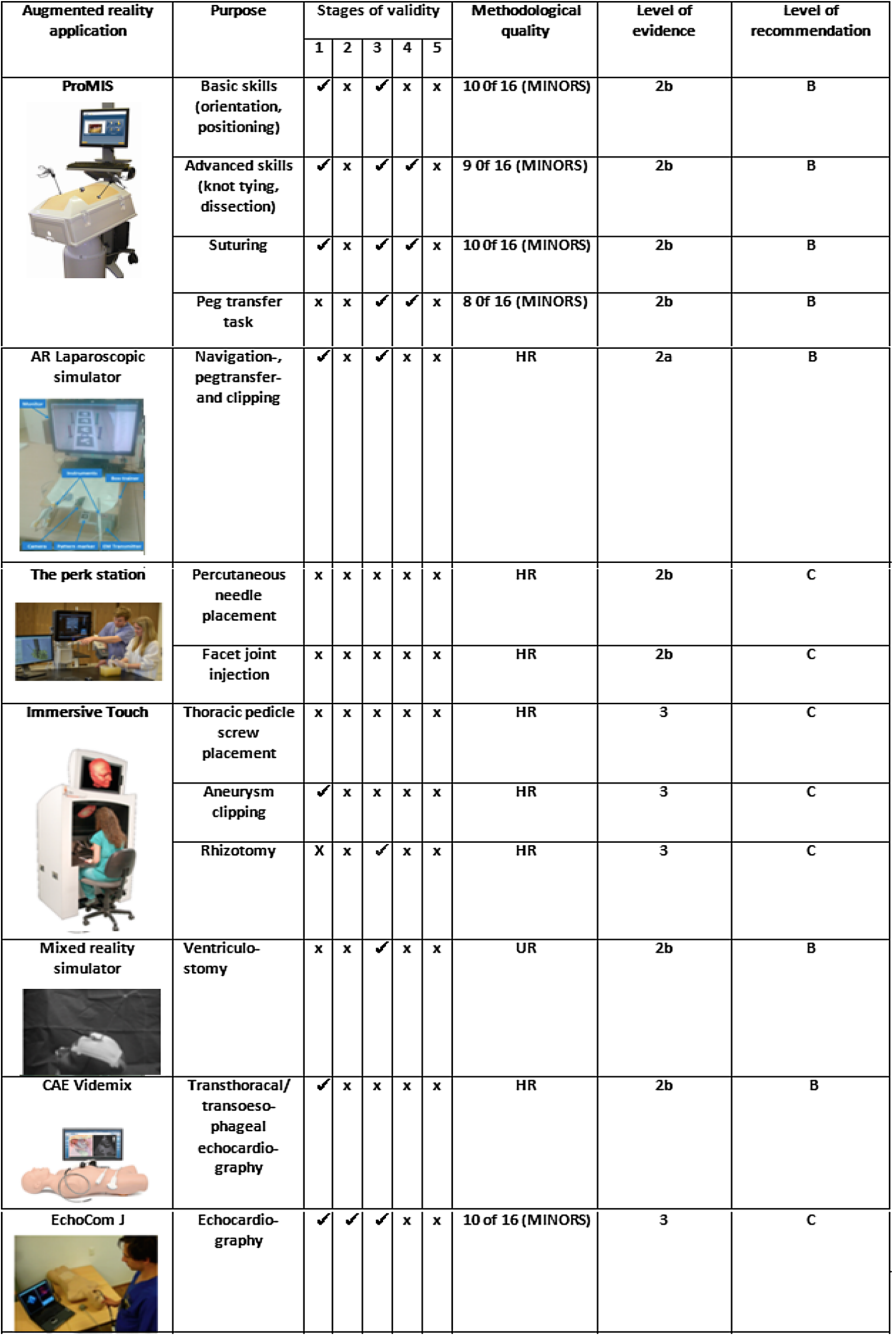
Overview of augmented reality applications (ARAs) and their methodological quality to train or educate medical professionals

The systematic search identified a total of 954 articles (Fig. 1). After removing duplicates, 767 articles were screened for relevance. Cross-reference search identified six more articles to be eligible. A total of 27 articles remained relevant for inclusion, describing seven ARAs used to train or educate medical professionals: the ProMIS Augmented Reality Simulator™, a laparoscopic simulator, the Perk Station, the Immersive Touch^®^, a Mixed Reality Ventriculostomy Simulator, EchocomJ and VIMEDIX™ (Fig. 2). ARAs were divided into three categories by educational purpose. Category 1 relates to ARAs used to train several tasks in laparoscopic surgery. Category 2 consists of applications used to train neurosurgical procedures. Category 3 describes ARAS of use in echocardiography. Other categories relating to purpose of use in training performance of medical professionals could not be retrieved.

Statistical pooling of data was not performed due to heterogeneity of study designs.

### Category 1: augmented reality application designed to train laparoscopic tasks

#### The ProMIS augmented reality simulator

The ProMIS is a simulator training laparoscopic procedures [21]. It contains an instrument tracking system, which captures instrument motion, while realistic haptic feedback is provided. Time, path length and smoothness of movement can be recorded objectively and used as outcome parameters. For these metrics, there is an intrinsic performance measurement, providing detailed information and statistics regarding a specific task. The systematic search identified thirteen studies assessing the use of the ProMIS augmented reality simulator (Haptica, Ireland) for training laparoscopic tasks including navigation, object positioning, suturing, knot tying and sharp dissection.

Botden and coworkers [33] tested face validity of the ARA using a questionnaire among 55 experienced and intermediate surgeons or surgical residents regarding realism, haptics and didactic value, comparing suturing and knot-tying performances. There was a general consensus considering ProMIS to be very realistic, with good haptics and a useful training tool, indicative for obtaining face validity.

Ten studies could be identified to provide evidence for construct validity of ProMIS [34–38]. Van Sickle et al. [37] demonstrated the apparatus’ ability to significantly distinguish between ten novice and experienced laparoscopists based on all parameters for a laparoscopic suturing task (*p* < 0.001). Nugent et al. [38] tested performance of 80 surgeons, surgical residents and students based on three basic laparoscopic modules. Experts outperformed postgraduate years (PGYs) 3 and 4, who in turn achieved better scores than the PGYs 1 and 2, who did better than the premedical students (*p* < 0.001). Results have shown that these differences between experience levels were significant based on all performance outcomes: time (*p* < 0.001), motion analysis (*p* < 0.001) and error score (*p* < 0.001), proving construct validity.

Overall, construct validity of ProMIS was established for outcome parameters time [34–38], path length [38] and smoothness of movement [36] comparing medical experts versus novices [36]. Results concerning validity were based on performance outcomes regarding navigation, object positioning, suturing, knot tying and sharp dissection.

Ritter et al. [39] tested 60 experienced, intermediates and novices. They established concurrent validity based on the comparison with the well-established FLS score for path length and smoothness with respect to the peg transfer task (*p* < 0.001). Botden and colleagues proved concurrent validity for the knot-tying task.

None of the reports considering ProMIS to train laparoscopic tasks investigated the instrument’s predictive validity.

#### AR laparoscopic simulator

Lahanas et al. [40] have developed a non-commercial AR laparoscopic simulator for training and assessment of surgical skills in minimally invasive surgery. Authors tested 20 experienced and novice surgeons. They provided evidence for face- and construct validity in all performance metrics for the instrument navigation-, peg transfer- and clipping task as the experienced group outperformed the novices significantly.

### Category 2: augmented reality applications designed to train neurosurgical procedures

#### The perk station

The Perk Station [41–43] is a training platform for image-guided interventions. While training on a phantom, trainees perform tasks using AR image overlay. The Perk Station intrinsically measures total procedure time, time inside phantom, path length, potential tissue damage, out-of-plane deviation and in-plane deviation. The Perk Station has been used to train facet joint injections and lumbar puncture.

None of the authors reported assessment of a validation process.

Two other studies used the Perk Tutor to investigate the effectiveness to train facet joint injections. By means of a randomized controlled trial, the value of the Perk Station in the learning process of percutaneous facet joint injections was assessed. The success rate of facet joint injections of the Perk Tutor trained group was significantly higher in comparison with the control group (*p* = 0.031), while potential tissue damage was significantly lower [41]. Time, time inside phantom, path inside phantom, out-of-plane deviation and in-plane deviation revealed no significant differences between the two groups [42].

Another study assessed twenty-four neurosurgical residents, randomly assigned to perform lumbar punctures using the Perk Station or without. Participants in the Perk Station group outperformed the control group by operating within a shorter distance (*p* = 0.02), a shorter period of needle insertion time (*p* = 0.05) and with less tissue damage compared to the control group (*p* = 0.01) [43].

#### The immersive touch augmented virtual reality system

The *immersive* touch augmented virtual reality system (IT) contains an electromagnetic head-tracking system in combination with a half-silvered mirror [44–46]. Outcome parameters of study are performance accuracy measurement and failure rate measurement. The device is described as a learning tool for training thoracic pedicle screw placement, clipping aneurysms and trigeminal rhizotomy.

Luciano et al. [44] used this system to train thoracic pedicle screw placement. The objective was to assess learning retention. Validity testing was not mentioned. The error rate was consistent with clinical results reported in the literature.

Seventeen neurosurgery residents used the IT to clip aneurysms. It was perceived as a useful educational tool by 64 % of the participants, while 71 % thought the simulator would help define which approach should be used in order to access the aneurysm safely, indicating face validity [45].

During a percutaneous trigeminal rhizotomy simulator session, seventy-one residents were divided into two groups based on experience. Increasing level of experience was significantly associated with a decreased distance from the ideal entry point (*p* = 0.001), a shorter distance from the target (*p* = 0.05) and a higher final score (*p* = 0.05), except for number of fluoroscopy shots (*p* = 0.52), indicative of construct validity [46].

#### A mixed reality ventriculostomy simulator

A third simulator, a novel mixed reality ventriculostomy simulator was described by Hooten et al. [47]. This simulator can be used as a training tool for a ventriculostomy procedure. In their study, 260 residents were divided in four groups based on experience. Use of the simulator was perceived as beneficial in training residents because of its realism. There was a general opinion the simulator would increase patient safety, both indicative for face validity. Senior and junior residents outperformed interns (*p* = 0.003). However, senior residents did not significantly outperform junior residents, making the achievement of construct validity questionable.

### Category 3: augmented reality applications used to train echocardiography

#### The CAE VIMEDIX™ ultrasound simulator

The CAE VIMEDIX™ ultrasound simulator uses a transducer, which provides positional and orientation data to reconstruct images in relation to a mannequin [48]. The simulator has been used to train transthoracic echocardiography (TTE) and transesophageal echocardiography (TOE).

The majority of the attendees claimed that the simulator was highly realistic (90 % agreed or strongly agreed for the TOE simulator and 87 % for the TTE simulator), proving face validity. These results were based on a questionnaire obtained from cardiology registrants and sonography students. Other forms of validity were not reported, nor an intrinsic experiment assessing specific performance skills.

#### The EchoCom

The EchoCom consists of a mannequin attached to a 3D tracking system and is used to train identifying congenital heart diseases based on sonographic information. Weidenbach et al. [49] tested 43 experts, intermediates and beginners. Face validity was proven as participants judged the simulator as realistic and useful. Evidence of content validity was achieved as experts evaluated the content of the simulator positively. Experts had a performance grade of 0.98, and intermediates and beginners had a mean value of 0.69 and 0.44, respectively. As all groups differed significantly in their diagnostic performance, construct validity was achieved.

## Discussion

Augmented reality applications (ARAs) are innovations wanting to be explored yet waiting to be scrutinized in medical education. The systematic literature review retrieved seven AR applications that have been developed in the field of medical professional training. AR allows trainees to understand the spatial relationships and concepts, and it provides substantial, contextual and situated learning experiences. Several of these ARAs can be viewed as a valid and reliable method for training. Moreover, AR helps to create authentic simulated experiences. It is thought to increases trainees’ subjective attractiveness, enhancing learning retention and performance. This is the first study to scrutinize the value of ARAs as a potential addition to the toolbox of medical professional education.

In modern times, the use of digital strategies to teach healthcare professionals has led to a major paradigm shift now reflected in many educational curricula [20, 50]. Computerized simulation models, mannequins and virtual reality simulators are used in medical professional training for partial-task rehearsal, full procedure rehearsal and team training. Studies that assessed the effect of simulation have shown a marked increase in self-reported confidence and comfort, technical skills and knowledge [51–53]. Furthermore, the transfer of skills to reality has been reported.

One of the limitations of VR simulation is that it has to render a full representation of the construct, which often leads to compromises because of costs and technical difficulties. Therefore, it may lead to rejection by (a part of) the trainees and educators. VR simulation in laparoscopic surgery has therefore only been applied as partial-task trainers [54].

Augmented reality differs from virtual reality in their ability to combine a physical simulation (such as laparoscopy equipment or mannequins) with a virtual reality overlay simulation, creating a truly immersive experience. Rare or complex situations, such as anatomical variations or emergencies, may be trained more optimally and realistically. This gives the opportunity for simulation training to transcend from partial-task training (such as laparoscopic dexterity exercises) to realistic full-task trainers that cover both interaction and complex spatial orientation (such as neurosurgery or echocardiography).

According to Gartner’s most current estimations, within 5- to 10-year AR, it is believed to have significant impact on society. Therefore, one needs to consider AR in the medical educational field seriously [55]. New commercially available technology such as Microsoft Hololens [56] Oculus Rift [57] and Google Cardbox [58], among others, is expected to propel new initiatives in medical training and education [56–59]. Medical educators should seek potential use, whilst remaining critical among their limitations. Only then will ARAs be a useful addition to medical training.

Our systematic search identified seven ARAs in the literature to date, designed to train medical professionals and professionals to be in institutionalized settings. Due to omit or the improper use of relevant keywords, it is possible that relevant articles were not within the range of search of this study. Although additional articles deemed relevant were found through cross-referencing, this might be the reason for an incomplete overview of all ARAs described in the literature.

The importance of validating new tools within the field of medical education is noted and illustrated by the fact that within all categories, validity steps have indeed been undertaken, especially since 2011. However, no follow-up studies on retention of skills could be identified, nor could subsequent clinical improvement of trainees be retrieved from studies. As no full validation strategies were outlined, it is unclear whether innovations assessed are of true value in training healthcare professionals. To date, it is unclear if the use of ARAs in training medical professionals is likely to contribute to patient safety. However, as training methods become more engaging and reliable, learning curves may be expected to become steeper and patients will ultimately benefit.

The main focus of surgical curricula has been on the acquisition of technical skills. However, to date, no surgical training methods have been developed to train residents how to avoid making errors during surgery. Training situational awareness should be essential, as errors result from misperceptions and using suboptimal problem-solving strategies [60]. Modern operating theaters are enriched with an enormous increase in new technology. This increases incoming signals and thus the mental load while performing surgery. AR allows the transfer of digital information into the real world, therefore blending two worlds together. In turn, this creates opportunities to filter input from the environment because additional information is within the surgeons’ field of vision. The use of AR is therefore preeminently suited for training curricula aiming at situational awareness. It is known that training situational awareness in high-risk environments such as the operating room is much needed, but lacking in medical educational curricula [61]. The benefit of AR could be widespread, from training better surgeons to making fewer errors in the operating room, ultimately leading to improvement of patient safety.

AR is a new technology in educational methodology. It has survived the initial phase and has shown the enormous potential within the medical field. Without doubt, healthcare will be profoundly affected developments in AR. As with any innovation, however, it is important to assess true value and place for results to be generated and curricula to sustain. Several applications have shown the potential of ARAs to bridge the gap between achieving the actual competence needed in the real working environment and training them in a virtual context. In order to implement existent and new ARAs in a training curriculum of medical specialists validly and reliably, uniform assessment strategies and complete validation trajectory are much needed. Only then, augmented reality training in medicine will become a winner in the digital revolution.

## References

[1] Vanderbilt AA, Grover AC, Pastis NJ, Feldman M, Granados DD, Murithi LK, Mainous AG (2014). Randomized controlled trials: a systematic review of laparoscopic surgery and simulation based training. Glob J Health Sci.

[2] Bharathan R, Vali S, Setchell T, Miskry T, Darzi A, Aggarwal R (2013). Psychomotor skills and cognitive load training on a virtual reality laparoscopic simulator for tubal surgery is effective. Eur J Obstet Gynecol Reprod Biol.

[3] Grantcharov TP, Kristiansen VB, Bendix J, Bardram L, Rosenberg J, Funch-Jensen P (2004). Randomized clinical trial of virtual reality simulation for laparoscopic skills training. Br J Surg.

[4] Schout BM, Ananias HJ, Bemelmans BL, d’Ancona FC, Muijtjens AM, Dolmans VE (2010). Transfer of cysto-urethroscopy skills from a virtual-reality simulator to the operating room: a randomized controlled trial. BJU Int.

[5] Schreuder HW, Oei G, Maas M, Borleffs JC, Schijven MP (2011). Implementation of simulation in surgical practice: minimally invasive surgery has taken the lead: the Dutch experience. Med Teach.

[6] Graafland M, Schraagen JM, Schijven MP (2012). Systematic review of serious games for medical education and surgical skills training. Br J Surg.

[7] Carmigniani J, Furht B, Anisetti M, Ceravolo P, Damiani E, Ivkovic M (2011). Augmented reality technologies, systems and applications. Multimed Tools Appl.

[8] Berryman DR (2012). Augmented reality: a review. Med Ref Serv Q.

[9] Guicherit OR (2015). The European working time directive and surgical residents’ expertise: no effect on the number of operations. Ned Tijdschr Geneeskd.

[10] Hopmans CJ, den Hoed PT, van der Laan L, van der Harst E, van der Elst M, Mannaerts GH, Dawson I, Timmand R, Wijnhoven BP, IJzermans JN (2015). Impact of the European working time directive (EWTD) on the operative experience of surgery residents. Surgery.

[11] Jamal MH, Wong S, Whalen TV (2014). Effects of the reduction of surgical residents’ work hours and implications for surgical residency programs: a narrative review. BMC Med Educ.

[12] Koh GC, Khoo HE, Wong ML, Koh D (2008). The effects of problem-based learning during medical school on physician competency: a systematic review. CMAJ.

[13] Khoiriyah U, Roberts C, Jorm C, van der Vleuten CP (2015). Enhancing students’ learning in problem based learning: validation of a self-assessment scale for active learning and critical thinking. BMC Med Educ.

[14] Burgess AW, Ramsey-Stewart G, May J, Mellis C (2012). Team-based learning methods in teaching topographical anatomy by dissection. ANZ J Surg.

[15] Burgess AW, McGregor DM, Mellis CM (2014). Applying established guidelines to team-based learning programs in medical schools: a systematic review. Acad Med.

[16] Jayakumar N, Brunckhorst O, Dasgupta P, Khan MS, Ahmed K (2015). e-Learning in Surgical Education: a Systematic Review. J Surg Educ.

[17] Pinto A, Brunese L, Pinto F, Acampora C, Romano L (2011). E-learning and education in radiology. Eur J Radiol.

[18] Wang X, Dunston PS (2007). Design, Strategies, and Issues Towards an Augmented Reality-based Construction Training Platform. ITcon.

[19] Wu H, Lee S, Chang H, Liang J (2013). Current status, opportunities and challenges of augmented reality in education. Comput Educ.

[20] Kamphuis C, Barsom E, Schijven M, Christoph N (2014). Augmented reality in medical education?. Perspect Med Educ.

[21] Botden SM, Jakimowicz JJ (2009). What is going on in augmented reality simulation in laparoscopic surgery?. Surg Endosc.

[22] Chu MW, Moore J, Peters T, Bainbridge D, McCarty D, Guiraudon GM (2012). Augmented reality image guidance improves navigation for beating heart mitral valve repair. Innovations (Phila).

[23] Low D, Lee CK, Dip LL, Ng WH, Ang BT, Ng I (2010). Augmented reality neurosurgical planning and navigation for surgical excision of parasagittal, falcine and convexity meningiomas. Br J Neurosurg.

[24] Szabo Z, Berg S, Sjokvist S, Gustafsson T, Carleberg P, Uppsall M (2013). Real-time intraoperative visualization of myocardial circulation using augmented reality temperature display. Int J Cardiovasc Imaging.

[25] Breton-Lopez J, Quero S, Botella C, Garcia-Palacios A, Banos RM, Alcaniz M (2010). An augmented reality system validation for the treatment of cockroach phobia. Cyberpsychol Behav Soc Netw.

[26] Lamounier E, Lopes K, Cardoso A, Andrade A, Soares A (2010). On the use of virtual and augmented reality for upper limb prostheses training and simulation. Conf Proc IEEE Eng Med Biol Soc.

[27] Mousavi HH, Khademi M, Dodakian L, Cramer SC, Lopes CV (2013). A Spatial Augmented Reality rehab system for post-stroke hand rehabilitation. Stud Health Technol Inform.

[28] Gallagher AG, Ritter EM, Satava RM (2003). Fundamental principles of validation, and reliability: rigorous science for the assessment of surgical education and training. Surg Endosc.

[29] Schijven MP, Jakimowicz JJ (2005). Validation of virtual reality simulators: key to the successful integration of a novel teaching technology into minimal access surgery. Minim Invasive Ther Allied Technol.

[30] Van Dongen KW, Tournoij E, van der Zee DC, Schijven MP, Broeders IA (2007). Construct validity of the LapSim: can the LapSim virtual reality simulator distinguish between novices and experts?. Surg Endosc.

[31] Higgins JPT, Green S (ed) (2011) Cochrane Handbook for Systematic Reviews of Interventions, version 5.1.0 [updated March 2011]. The Cochrane Collaboration http://www.cochrane-handbook.org/. Accessed 26 Jan 2016

[32] Slim K, Nini E, Forestier D, Kwiatkowski F, Panis Y, Chipponi J (2003). Methodological index for non-randomized studies (minors): development and validation of a new instrument. ANZ J Surg.

[33] Botden SM, Berlage JT, Schijven MP, Jakimowicz JJ (2008). Face validity study of the ProMIS augmented reality laparoscopic suturing simulator. Surg Technol Int.

[34] Broe D, Ridgway PF, Johnson S, Tierney S, Conlon KC (2006). Construct validation of a novel hybrid surgical simulator. Surg Endosc.

[35] Gilliam AD (2009). Construct validity of the ProMIS laparoscopic simulator. Surg Endosc.

[36] Pellen MG, Horgan LF, Barton JR, Attwood SE (2009). Construct validity of the ProMIS laparoscopic simulator. Surg Endosc.

[37] Van Sickle KR, McClusky DA, Gallagher AG, Smith CD (2005). Construct validation of the ProMIS simulator using a novel laparoscopic suturing task. Surg Endosc.

[38] Nugent E, Shirilla N, Hafeez A, O’Riordain DS, Traynor O, Harrison AM (2013). Development and evaluation of a simulator-based laparoscopic training program for surgical novices. Surg Endosc.

[39] Ritter EM, Kindelan TW, Michael C, Pimentel EA, Bowyer MW (2007). Concurrent validity of augmented reality metrics applied to the fundamentals of laparoscopic surgery (FLS). Surg Endosc.

[40] Lahanas V, Loukas C, Smailis N, Georgiou E (2015). A novel augmented reality simulator for skills assessment in minimal invasive surgery. Surg Endosc.

[41] Moult E, Ungi T, Welch M, Lu J, McGraw RC, Fichtinger G (2013). Ultrasound-guided facet joint injection training using Perk Tutor. Int J Comput Assist Radiol Surg.

[42] Keri Z, Sydor D, Ungi T, Holden MS, McGraw R, Mousavi P (2015). Computerized training system for ultrasound-guided lumbar puncture on abnormal spine models: a randomized controlled trial. Can J Anaesth.

[43] Yeo CT, Ungi T, Thainual P, Lasso A, McGraw RC, Fichtinger G (2011). The effect of augmented reality training on percutaneous needle placement in spinal facet joint injections. IEEE Trans Biomed Eng.

[44] Luciano CJ, Banerjee PP, Bellotte B, Oh GM, Lemole M, Charbel FT (2011). Learning retention of thoracic pedicle screw placement using a high-resolution augmented reality simulator with haptic feedback. Neurosurgery.

[45] Alaraj A, Luciano CJ, Bailey DP, Elsenousi A, Roitberg BZ, Bernardo A (2015). Virtual reality cerebral aneurysm clipping simulation with real-time haptic feedback. Neurosurgery.

[46] Shakur SF, Luciano CJ, Kania P, Roitberg BZ, Banerjee PP, Slavin KV (2015). Usefulness of a Virtual Reality Percutaneous Trigeminal Rhizotomy Simulator in Neurosurgical Training. Neurosurgery.

[47] Hooten KG, Lister JR, Lombard G, Lizdas DE, Lampotang S, Rajon DA, Bova F, Murad GJ (2014). Mixed reality ventriculostomy simulation: experience in neurosurgical residency. Neurosurgery.

[48] Platts DG, Humphries J, Burstow DJ, Anderson B, Forshaw T, Scalia GM (2012). The use of computerised simulators for training of transthoracic and transoesophageal echocardiography. The future of echocardiographic training?. Heart Lung Circ.

[49] Weidenbach M, Razek V, Wild F, Khambadkone S, Berlage T, Janousek J, Marek J (2009). Simulation of congenital heart defects: a novel way of training in echocardiography. Heart.

[50] Rouch JD, Wagner JP, Scott A, Chen DC, DeUgarte DA, Shew SB, Tilou A, Dunn JC, Lee SL (2015). Innovation in pediatric surgical education for general surgery residents: a mobile web resource. J Surg Educ.

[51] Fernandez GL, Page DW, Coe NP, Lee PC, Patterson LA, Skylizard L (2012). Boot cAMP: educational outcomes after 4 successive years of preparatory simulation-based training at onset of internship. J Surg Educ.

[52] Peyre SE, Peyre CG, Sullivan ME, Towfigh S (2006). A surgical skills elective can improve student confidence prior to internship. J Surg Res.

[53] Zeng W, Woodhouse J, Brunt LM (2010). Do preclinical background and clerkship experiences impact skills performance in an accelerated internship preparation course for senior medical students?. Surgery.

[54] Schreuder HW, Oei G, Maas M, Borleffs JC, Schijven MP (2011). Implementation of simulation in surgical practice: minimally invasive surgery has taken the lead: the Dutch experience. Med Teach.

[55] Gartner http://www.gartner.com/newsroom/id/3114217. Accessed 26 Jan 2016

[56] Engadget http://www.engadget.com/2015/07/08/microsoft-hololens-medical-student-demo/. Accessed 26 Jan 2016

[57] Forbes http://www.forbes.com/forbes/welcome/. Accessed 26 Jan 2016

[58] http://www.imedicalapps.com/2015/06/google-cardboard-apps-youtube-360o-impact-medicine/. Accessed 26 Jan 2016

[59] Schreinemacher MH, Graafland M, Schijven MP (2014). Google glass in surgery. Surg Innov.

[60] Guze AP (2015). Using Technology to Meet the Challenges of Medical Education. Trans Am Clin Climatol Assoc.

[61] Graafland M, Schraagen JM, Boermeester MA, Bemelman WA, Schijven MP (2015). Training situational awareness to reduce surgical errors in the operating room. Br J Surg.

